# Factors Affecting the Initial Engagement of Older Adults in the Use of Interactive Technology

**DOI:** 10.3390/ijerph18062847

**Published:** 2021-03-11

**Authors:** Lina Lee, Mary Lou Maher

**Affiliations:** Department of Software and Information Systems, University of North Carolina at Charlotte, Charlotte, NC 28223, USA; m.maher@uncc.edu

**Keywords:** engagement, older adults, technology, empirical studies

## Abstract

Smart environments and the use of interactive technology has the potential to improve the quality of life for the senior community as well as to support the connections among the senior community and the world outside their community. In addition to the increasing number of studies in the field of aging and technologies, research is needed to understand the practical issues of user focus, adoption, and engagement for older adults to accept interactive technologies in their lives. In this study, we use two commercial technological interventions (uDraw and GrandPad) to understand technology-related perceptions and behaviors of older adults. We present five case studies that emerge from empirical observations of initial engagement with technology through research methods such as focus group discussions, in-depth interviews, observations, and diary studies. The contributions of this study are identification of the key factors that influence the initial engagement with interactive technology for older adults.

## 1. Introduction

World demographics have shifted in the last twenty years by the drop in the birth rates and an upward shift in life expectancy [1]. As a result, there is a significant rise in the number of older people. Along with this rise in the number of older people, recent years have shown a large rise in the use of technology in all aspects of daily life. Many researchers believe that the application of technology to the aging process can help people “age well” and stay active [2,3]. Various technological inventions have been developed for older adults to facilitate independence when performing essential activities as well as to stay connected with family members and friends [4,5]. Technological literacy is increasingly required to live for the senior community [6] because older adults will be expected to use interactive technologies in their living environments, regardless of their willingness or not to engage with technology [7]. In particular, the COVID-19 pandemic has triggered an urgent need to address societal changes of an aging population due to social isolation and the need for health care [8,9]. This results in an increased demand for the design and adoption of interactive technologies to assure the health of older adults.

This paper has a focus on the need to build an in-depth understanding of older adults’ perception and preferences towards technology. Despite the increasing amount of studies in the field of aging and technologies [10,11], not enough research has been done to understand the practical issues of engagement in accepting interactive technologies by older adults. Many studies tend to focus on evaluating the feasibility or usability of technology [12,13,14,15]. Recent research about aging and technology tends to underestimate the initial barrier to use technology faced by older adults.

In this study, we address this need by asking the overarching research question: What are the key factors that engage the older population in the use of technology to adapt and live well in the digitized world? We study a senior community and their responses to the use of specific interactive technologies to understand technology-related perceptions and behaviors of older adults. The goal of this study is to identify the key factors that influence the engagement of older adults in the adoption of interactive technology. To achieve this, we took a more holistic approach, engaging with over 50 people, including multiple stakeholders over six months: program director and staff members in the senior community center, social workers, and volunteers for the activities that provided in the senior center, family members, relatives, or religious group. 

Our methodology is a qualitative ethnographic study in which the researcher directly observes and evaluates older adults’ technological behavior in the research context [16]. We use data accumulated over six months through research methods such as focus group discussions, in-depth interviews, observations, and a diary study using two commercial products (uDraw (https://en.wikipedia.org/wiki/UDraw_GameTablet (accessed on 14 November 2010))) and GrandPad (https://www.grandpad.net/) (accessed on 1 May 2018). We conduct an inductive thematic analysis on the multiple sources of data [17]. From our analysis, five case studies have emerged that categorically describe the variety of attitudes towards interactive technologies: (1) active towards technology, (2) passive towards technology, (3) social use of technology, (4) diverse use of technology, and (5) family oriented use of technology. Based on these cases, we present new engagement factors that emerge from our empirical observations of older adults and their response to interactive technology. 

## 2. Background

In this section, we show that the focus of research on the use of technology by older adults tends to be about the limitations of this population and the impact of the limitations on the design and usability of the technology. In contrast, our study has a focus on the attitudes, perceptions, and engagement of older adults with technology. We also review the methodologies for research on interactive technologies for older adults and recognize a need for ethnographic methods that can provide more in-depth studies of individuals in situ rather than methods that generalize across large numbers of participants in laboratory settings.

### 2.1. Current Research Focus for Older Adults’ Use of Technology

Many studies have been conducted to understand the challenges and barriers that older adults face and the factors that affect their adapting to new technologies and their continued use. However, there is a relatively little research exploring older adults’ initial and long-term engagement with interactive technology.

In order to design appropriate interactive technology for older adults, understanding aging limitations are important [18,19,20]. When people start aging, their physical and cognitive abilities tend to reduce slowly and gradually end, which in turn forces them to start developing negative attitudes towards interactive technologies [21]. Older adults may have doubts that technological innovations are real and become nervous since they are not confident in their ability to manage and practically adopt these innovations [22]. The mistrust of technology may escalate to technophobia, which makes older adults less likely to try out new technology [23,24,25]. Peek et al. [22] explain that technophobia is normal for people who did not grow up in environments that are populated by digital systems and changing mindset does not happen quickly. To support older adults, service providers, researchers, designers, developers, and policymakers have long been working together to better understand how older adults can achieve optimal health and wellbeing with the benefit of technology [26]. 

Other studies have focused on factors that affect adoption of technology by older adults and its continued usage. Many scholars show a lower level of innovative technology adoption by older adults [27,28,29,30]. To make interactive technologies more acceptable, the notions of usability [31,32], independence, convenience [33,34], devices to support physical activity [35,36], technology training and education [37,38], the role of caregiver [39,40], innovation (focus on developing new features) [41,42,43], affordability [44,45] and potential or continued benefit [28,29] have been explored by many scholars over the past decades. These studies focus on technologies that are usable or adoptable and suitable for continued use, but they typically do not focus on providing pleasurable and positive experiences to encourage initial and long-term engagement. 

Older adults’ engagement and enthusiasm are key elements in determining the success in their use of interactive technology. By reviewing the literature, we learn that despite the barrier to adoption being the most difficult stage, there is a lack of research for understanding older adults’ initial engagement. Due to age-related factors, older people often have difficulty overcoming the initial barrier to use interactive technology [46,47]. The significant problem in the use of technology by older adults is that even though a broad and advanced range of tools and platforms for older adults are being developed, the number of older adults who actively utilize the technology remain few. Therefore, our research explores the complex phenomena of human values to understand the essential behavioral characteristics and identify the key factors that influence the initial engagement of older adults with technology.

### 2.2. Current Research Challenges for Older Adults

There are methodological and design challenges in studying older adults [48], including recruitment, informed consent, reliable responses, communication with older adults, and providing appropriate instruction about research. Older adults may fear that refusing to participate in a study. The number of participants may affect the quality and validity of the finding of the study [49]. According to Birkland [48], many researchers conduct user studies with older adults that rely on self-report methods. Empirical studies that use a mixed-method design are rare. The main problem of short-term surveys or interviews is that these rely on users’ recollection and self-interpretations. Older adults tend to provide responses to researchers that are more positive or more frequent than in reality. Results show that older adults often assess the technology as positive, however, they fail to show clear evidence that older adults will adopt new emerging technology into their lives [50,51]. Many studies heavily focus on testing the usability by older adults with a lab study over a short period of time [52,53,54]. 

The vast majority of the research use age as the sampling criteria [48]. In many studies, older adults are often considered as a homogeneous group in technology research studies regardless of their characteristics, abilities, or technology experiences [55,56]. However, older adults are heterogeneous, and it is difficult to generalize the characteristics of technology use due to various factors such as physical, psychological, emotional, social, and economic situations of older adults [57]. This research explores the behavioral characteristics of older adults when asked to initiate the engagement of new technology rather than to evaluate the usability of new technology. Instead of focusing on a specific target demographic who need to supplement their physical ability, we focus on broad engagement themes as a starting point in studying older adults’ engagement towards technology. This study conducts an ethnographic study to understand the complex phenomena surrounding older adults which enable researchers and designers to predict and provide the processes to develop engaging technologies for older adults [13]. 

## 3. Methodology for Studying the Factors That Affect Older Adults’ Initial Engagement with Technology

The methodology for this study includes choosing the technology interventions and using a mixed methods approach that includes ethnographic observational data. We explain the reason for choosing commercial products for the intervention. Then, we describe the mixed methods studies specific to our research goal of identifying the factors that increase older adults’ initial interest and their subsequent engagement with interactive technology.

### 3.1. Technological Intervention

Initially, we conducted a pilot study with five senior residents living in a retirement community to learn about the factors that may stimulate their interest in the use of technology [58,59]. The main topic of the discussion was about activities that interest older adults and how to spend leisure time. We found that many older adults enjoy being creative, playing games, and being social. With the results, we decided to focus on two user experiences using interactive technology: (1) social creative expression in public space, and (2) emotional attachment in a private setting.

When selecting the interactive technology for the longitudinal study reported in this paper, we chose two commercial products as the context in which we observed and collected data so that we could reduce the issues related to poor usability that occur in technologies developed by research students. The uDraw Game Tablet is an embodied interactive technology that facilitates creative social expression by allowing users to create free form drawings, artwork, and games. GrandPad is a communication technology that enables older adults to easily communicate with their loved ones. In the context of a longitudinal study, older adults may lose interest quickly if they experience difficulty in understanding how to use the system. We wanted to avoid disinterest due to using a prototype system that might have unexpected usability issues or is an unstable implementation. If the purpose of this study was to propose a new practical solution to older adults with a specific problem through technology, the user-related study would have been carried out using technology designed on our own. However, since this research is not intended to evaluate the feasibility of technology or focus on technological solutions but to observe cases in which older adults are engaged when experiencing technology, commercial products were used. 

### 3.2. Mixed Methods Studies

Our methodology includes participant observation, augmented by a range of other approaches: the use of focus groups, semi-structured interviews, participant diaries. Our findings are based on interacting with older adults in the context of previous research and the accumulated experience of the researcher. 

#### 3.2.1. uDraw Study of Initial Engagement in a Community Center

##### Observation (with Facilitator)

Participant observation is a central data collection method in the uDraw study. We collected data at a shared community room at a senior residence. The researcher visited the site for three hours each visit, three times a week, over three months. We recorded the activities, participant attitudes or behaviors, and noted relevant details. To observe senior residents effectively, we operated a help desk to engage in participant observation to gain insight into the culture of the community of older adults. Significant time and effort were given to open-ended conversations with residents. We offered training in the uDraw system and provided personal help for older adults to learn mobile apps, web search, and other appliances that tailor to their interests. Although there were about 40 residents in the community we observed, we used the data from 19 residents that were most involved in the technology interventions and help desk support activities. Of the 19 residents, 11 experienced the uDraw system with the help of researchers; 8 residents made use of the help desk (7 males and 1 female); 3 out of the 8 visited the help desk repeatedly with the main purpose to learn mobile apps.

Video recorded data is used to observe the natural behavior around the system when a facilitator is not located. The uDraw system was constantly running in a shared community room throughout the three month research period. Residents were free to try and use the uDraw system. Two video recording devices (Google Nest Cam) were located near the screen to capture the study area. One was directed toward the user and the tablet, to record all behaviors occurring when the system was used, while the other was toward the public large display, to record social behaviors around the system. The motion activated cameras would turn on and start recording when they detected motion. This study was approved by the Institutional Review Board (IRB) to collect data from people who walked down the hall or past the interaction area but who had no intention of interacting. We collected video data over 92 days. We segmented this data so we could identify data referring directly to the usability of the uDraw system, referring to content about hobbies or interests of older adults, or referring to technology-related matters. This particular situation was separately extracted, and the issues were noted and discussed. Most residents watched TV while knitting, using a coffee machine, or using a copy machine, with 21 residents showing a repeating pattern. Of the residents, 12 appeared to be interested in activities provided by the community center and in using digital devices provided by volunteers. In the case of the uDraw system we provided, only one person was observed using the uDraw system voluntarily, and the number of times this individual used the uDraw system without the help of a researcher was 9 times.

We conducted in-person semi-structured interviews to understand general attitudes towards interactive technology with regard to 8 factors (positive affect, comfort, feel involved, perceived benefits and usefulness, controllability, help, discoverability and learnability, and persistence) as shown in Table 1. We interviewed 4 residents, and they also participated in the focus group discussions. All interviews were audio recorded and transcribed with the participants’ consent. While participant observation gives information on the action and behavior of older adults in the context of interactive technologies, interviews provide us with data on how people directly reflect on their own behavior, circumstances, identities, and events. 

The focus group discussions were conducted every two weeks, a total of five focus group discussions with four residents. We let participants play the uDraw Pictionary game before participating in the focus group discussion. This game is an art-based video game in which players can play on a uDraw Game Tablet. In uDraw Pictionary, the players refer to a particular subject to draw a picture. The teammates are then tasked with the challenge of guessing the words each image is supposed to portray. After one hour of play, we discussed the same 8 factors as the interview prompts used in the pre-interview questions (slightly modified) to gain insights about older adults’ engagement in the use of the given system. The focus group was audio recorded and transcribed.

#### 3.2.2. GrandPad Study of Initial Engagement in a Family Setting

The diary study is a central data collection method of the GrandPad study with a total of 3 older adults and family members. In this study, the older adults received one GrandPad tablet for three months. Family members could connect to GrandPad’s private family network via iPhone, Android phone, or desktop computer. They could manage the functions for older adults from the convenient companion app. The researcher purchased and created an account on the GrandPad app with a monthly subscription including the convenience of unlimited data. We gathered data from the usage log. The researcher collected usage records every day. Since the fact of recording their usage may affect their behavior; it was not mentioned to the older adults. We also gathered data from diary studies submitted by the family member for each older adult with approval from our institution’s IRB. There are a lot of limitations in conducting a diary study with older adults. Older adults feel writing a diary is an extra burden, so the likelihood of a negative impact on the use of a GrandPad device cannot be excluded. For this reason, the diary study was conducted with family members. Using mobile diary studies software, the participant wrote a diary about older adults’ GrandPad usage through a smartphone. One or two younger family members who are in the closest relationship with older adults played a role in sending a prompt to encourage older adults to use the GrandPad. The researcher sent journal prompts to family members through text or email at 9 am every day, however younger family members could create their prompts as well. The prompts were created in a way to encourage older adults to actively use the GrandPad App. These prompts were used for seniors to inspire them to be creative, share significant memories, and keep their minds healthy and active.

There was a significant difference in the frequency of GrandPad use of the three families. The first user was given the first GrandPad on 7 November 2019, and used it until 22 January 2020, and 73 diary entries were recorded, and the total usage time was 141 h. The second family started using it on 8 November 2019, but the last recorded date of using the GrandPad was 20 November, with the device only being used 12 times. The total usage time was 3 h, with 6 diary entries being recorded. The last family used it from 11 November to 5 January, and there were a total of 44 data entries, with a total usage time of 43 h.

We conducted in-person interviews with the GrandPad participants to understand general attitudes towards communication technology. We used the same interview questions as the uDraw study. 

### 3.3. Analysis

During the research period, we immersed ourselves in the senior community and spent time talking directly with the participants and observing their lives and attitudes towards technology. We conducted an inductive thematic analysis [17]. This qualitative analysis focused on: (1) categorizing older adults to present the diverse attitudes towards interactive technologies; (2) identification of the factors of initial engagement in the use of interactive technologies. We focus on finding situations wherein older adults show emotionally positive affect related to use or discussion about interactive technology and finding situations where older adults become active and motivated by a desire to physically try to use technology. Two researchers met weekly to discuss the collected data of all activities, reflecting and iterating over the themes that lead our discussions, until a consensus was found. With the written field notes, video data analysis, direct observations, diary written by participants, and qualitative data, we extracted 5 case studies and 9 initial engagement values. Below, we present the results of the analysis, organized by themes.

## 4. Five Case Studies That Emerge from Empirical Observations of Initial Engagement with Technology

A total of 36 subjects are classified into five categories: positive about technology, negative about technology, social use of technology, diverse use of technology, and family oriented use of technology. By observing each participant over three months (the length of observation varies depending on participants, approximately from 10 days to 3 months for each participant), we consider six factors to define five case studies: family relationships, social contacts, general attitudes towards technology, need for technology, physical and cognitive health, and motivation. 

### 4.1. Positive (Active) about Technology

**Figure 1:**
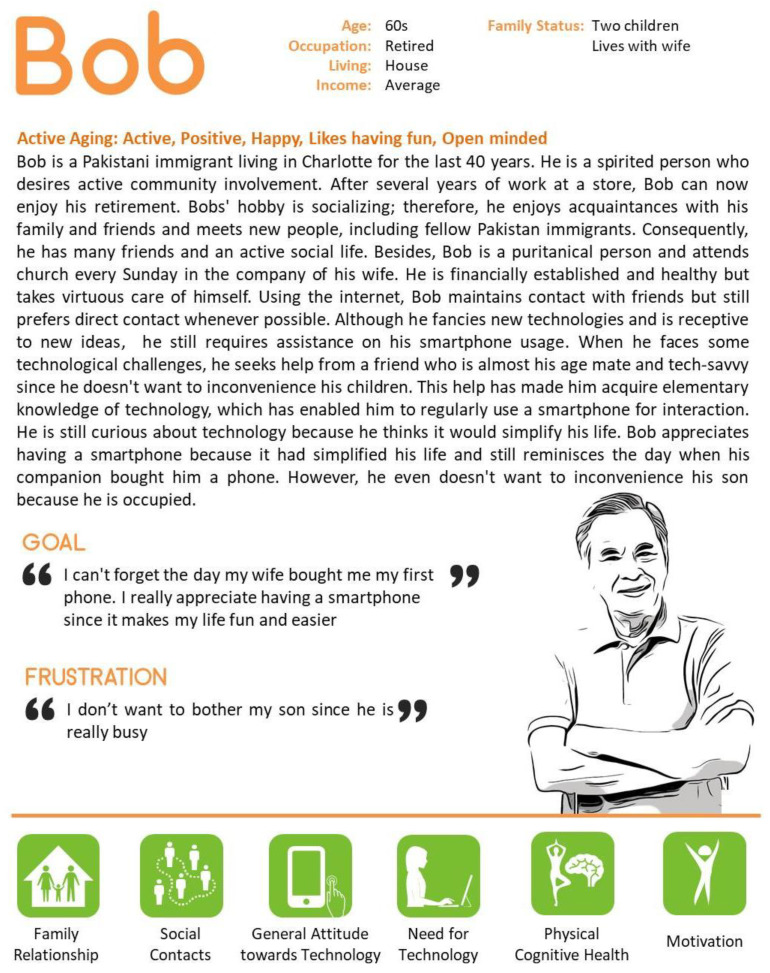
Case 1—Representative character to present positive attitude towards technology.

Bob is a participant in the GrandPad study. Out of the three participants of the GrandPad study, Bob was the only one who had no problems with trying something new or using new functions of mobile technology. In the Diary study, Bob faithfully performed the daily tasks given, and overall, he did not report any difficulty or inconvenience in using the system. The three apps that Bob was most interested in and used the most were the reading, game, and music apps of the GrandPad. Bob has maintained a positive attitude toward the phone from the moment he first owned the mobile phone. When responding to his experience with technology, Bob often connected his experiences and memories of technology with people (See quotation in Figure 1), which reminds him of positive memories of that moment about mobile phones rather than remembering functions related to mobile phones.

### 4.2. Negative (Passive) about Technology 

**Figure 2:**
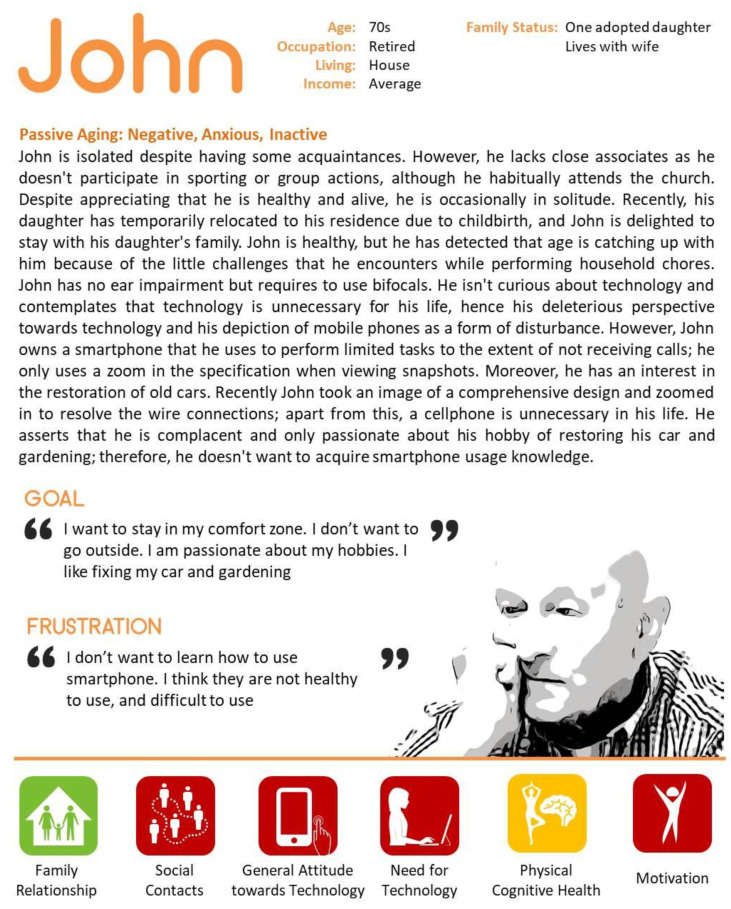
Case 2—Representative character to present negative attitude towards technology.

John was also a participant in the GrandPad study. John had a negative attitude toward using technology (See Figure 2). He was not very interested in using the GrandPad during the research period except for the time that he explored mobile technology for his hobbies. Out of the three participants, John’s total usage time was the lowest. He only used the GrandPad for 12 days in the first month of the study. Further, he did not complete the daily tasks very well, not because he faced technical difficulties but because he had no interest in the activities. However, the apps he used the most during the study were the magnifier app and the music app. John was relatively comfortable using the computer because he learned how to operate it when he was in the military before his retirement. John often loses interests and complains about issues related to physical discomfort. For example, the mobility of his finger has deteriorated, so that a button cannot be properly clicked, and he found it difficult to read the screen due to poor vision. John has a very negative attitude to mobile phones, despite being experienced in using the technology.

### 4.3. Family Oriented Use of Technology

**Figure 3:**
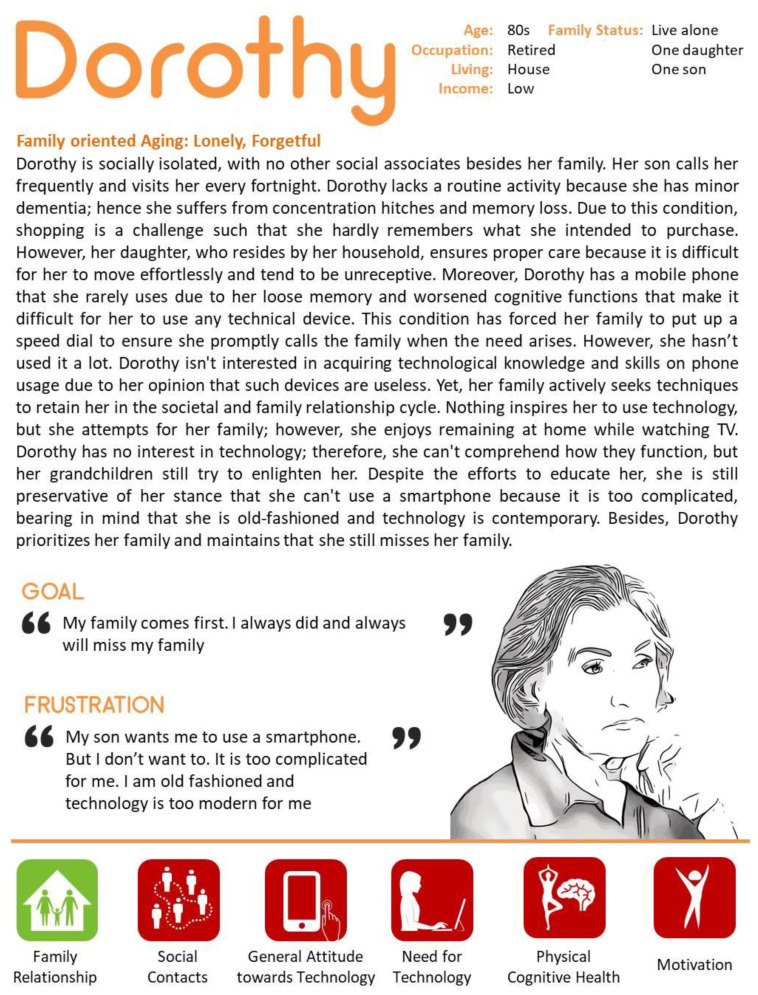
Case 3—Representative character to present family oriented use of technology.

Dorothy was the oldest of the participants in the GrandPad study. She was not able to use the GrandPad on her own and constantly needed her family’s help. She is an example case that shows how negative attitudes towards technology can be changed in a positive direction. With the support of her family, however, Dorothy made great strides in using the technology’s features during the study period. For her, family is an important factor in deciding to use technology (See Figure 3). 

### 4.4. Social Use of Technology

**Figure 4:**
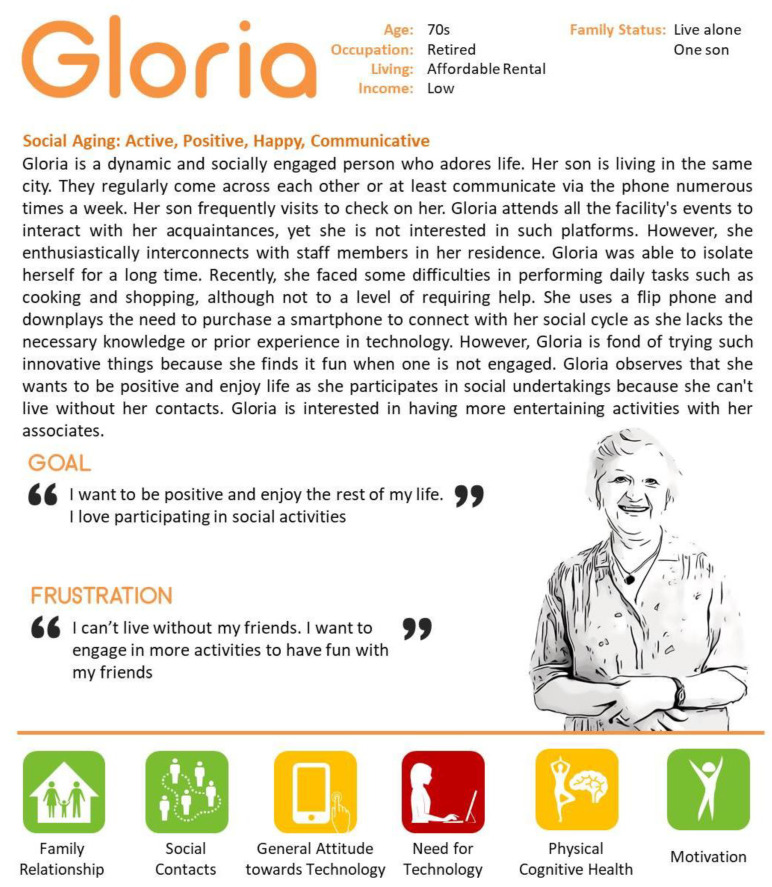
Case 4—Representative character to present social use of technology.

Gloria is described as a representative of the social use of technology, but two more participants named Peggie and Eva are also important in describing this case. All three were participants of the uDraw study. They participated in all five focus group discussions during the research period. Gloria visited the researcher at the designated time and additionally used the uDraw system twice. The other two participants did not use the system except during the focus group discussions and an initial training session. All three participants were not willing to use the system on their own, and the researcher’s support was always required. They expressed difficulty when using the uDraw tablet but followed the researcher’s instructions well. A characteristic of those described by this case is that they attend many activities at the community center to stay occupied (See Figure 4). In this case, we found that older adults’ motivation for using technology was to maintain social relationships in a community center because they have a strong desire to engage and sustain others’ interests.

### 4.5. Diverse Use of Technology

**Figure 5:**
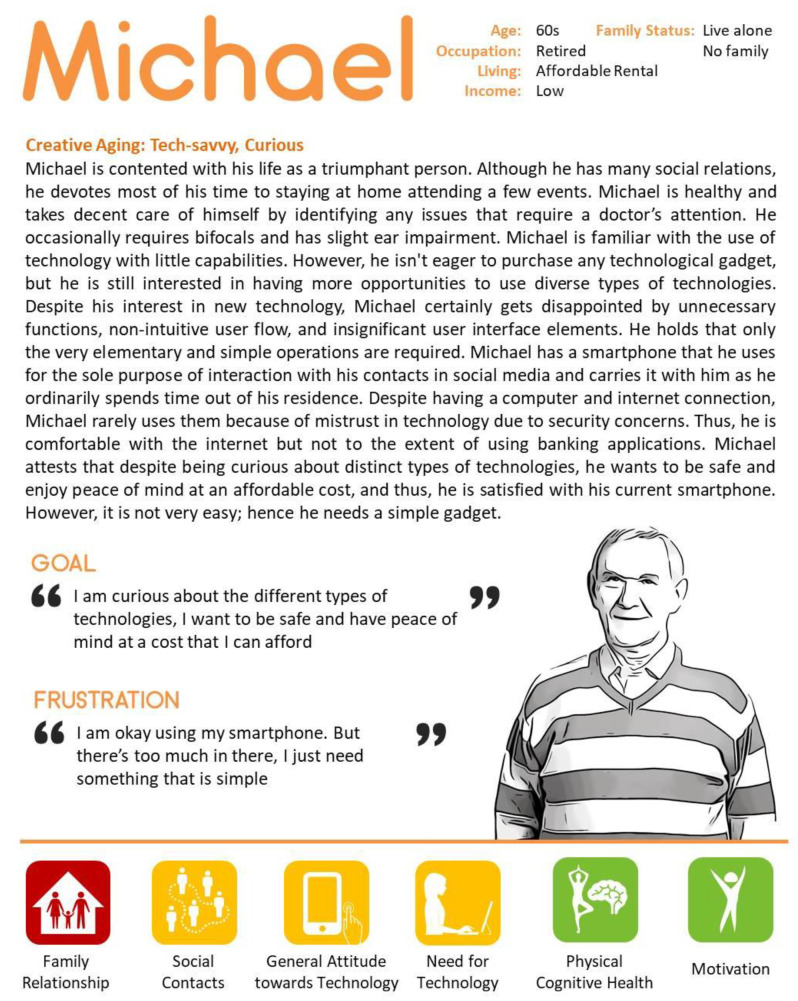
Case 5—Representative character to present diverse use of technology.

Michael was another participant in the uDraw study. He was the participant with the highest initial interest in the uDraw system. As in the case of Gloria, Peggie, and Eva, he also participated in five focus group discussions. In the beginning, he expressed high interest and engagement but could not sustain his engagement for three months. In the first month, he used the system by himself nine times. Of the four participants, Michael was the only one who used the system without any difficulty. He skillfully used the functions he had learned and experienced with the researcher, but he did not try new functions. Therefore, the range of functions that could be used was limited. He often visited the help desk and showed interests about new interactive technology such as Virtual Reality (VR), a voice-activated speaker, and a home robot (See Figure 5).

## 5. New Initial Engagement Values Emerging from Our Case Studies

In this section, we discuss new initial engagement values that have emerged from the five case studies. Under each factor, we compare to values that are discussed in existing literature as a way to support older adults’ use of technology. We establish the meaning of each factor from the views of the participants with an understanding of their context and behavior during the observational period. 

### 5.1. Motivation (Desirability)

#### Older Adults’ Motivation Needs to Be Considered before Usability to Increase Initial Engagement for Older Adults

Most scholars have indicated that usability is a critical issue to help older adults to learn how to use the interactive systems independently [28,52,53]. Davis [60] identified the ease of use for interactive technology as a significant determinant of the adoption for an individual older adult. However, our study identified desirability should be considered before usability for engaging older adults. Whereas usability influence user’s ability to complete a task [61], desirability means they have a pleasurable and engaging experience while using the system. Initial Engagement will increase when older adults have specific reasons or motivation to use interactive technology for having fun and engaging experiences. In the case of Dorothy, she does not like to use the mobile phone so usability was not the primary issue. When I asked about ‘What do you like best about your cell phone?’, Dorothy could not answer for a long time. Her daughter, Angela, encouraged her to answer.

Dorothy: *“I don’t really know how to answer you, if you want to know the truth…Back again to being old fashioned, I’m not modern. There are some things that I don’t understand. I put up with it, with help. Yeah, with help. I’ve had to rely on my son to guide me. And I don’t know if I’ll ever be much different from what I am now.”*

We observed her attitude changes after we delivered the GrandPad to Dorothy and analyzed her usage through a diary study. When she received the GrandPad for the first time, the only thing she did was turn on the GrandPad and watch the photos posted by her children and granddaughter. Dorothy’s children registered the Companion App to continue sending photos. Her family’s efforts enabled her to appreciate the daily life of her grandchildren. The following quotes are the feedback of a diary study from her family members.

*“Mom is still warming up to it. She looked at picture today”*

Dorothy needed help on how to use all the features except the ability to turn off the screen by herself. Five days after using GrandPad, she was able to send a text and picture through GrandPad with the help of Angela.

*“Mom sent her brother a text/email this morning thanking him for the pictures that he sent her. Even though it was just 1 sentence, it overwhelmed her, but we will get her there.”*

Sixteen days later (Nov, 28), we could see that Dorothy has finally figured out how to send a photo by herself. Then one day after (Nov, 29), we could get a response that she actually enjoys the GrandPad.

*“Mom did a good job posting a photo of the Thanksgiving dinner that my sister is preparing. She seems to be enjoying GrandPad”*

Dorothy, who has no interest in using technology and feels that it’s not a necessity in her life, comes to realize that it is possible to listen to any song she wants through technology a few days after using GrandPad. This case shows that Dorothy’s attention to her family’s photos using GrandPad leads to her motivation to learn how to listen to music on the Grandpad app. In the beginning of the Grandpad intervention, Dorothy could not use the app for a long time because it made her feel tired, but eventually she became motivated by family and then she was delighted to learn new features.

*“Mom just sent me an email informing me that Choo Choo Ch’ boogie by Louis Jordan is the song that she listened to today.”*

This case shows that desirability should be given priority over designing technology rather than usability in the early phase of the design. Dorothy was engaged to listen to music and therefore was able to focus on the usability of the technology. This encouraged her to learn to use other features like games and puzzles. 

### 5.2. Social 

#### Creating the Opportunity to Build Social Relationships with a Technology Need to Be Considered Rather Than Supporting the Ability to Being Alone for Older Adults

As people begin to grow older, older adults become passive to participate in activities that are critical for their wellbeing [62]. Interactive technologies such as “aging in place” and “assisted technologies’’ assist with such activities or support older adults who are deficient and in need of assistance to execute them to promote their independence [39,63,64]. These technologies are expected to be used mainly in situations where older adults are alone. However, our study shows that Initial Engagement will increase when there is more opportunity to build a social relationship while using interactive technology. 

We learn through the case of Gloria, Peggie, and Eva, how social relationships positively influence their interest in technology. Older adults show a preference for emotionally meaningful and positive relationships with others in the senior community [65,66]. Gloria enjoys investing in personal effort in keeping up her social relationships. We were able to see how socially active older individuals are changing the community. Gloria is a central part of social activities in the community. She constantly invites and encourages other residents to participate in activities provided by the community center. Looking at how Peggie and Eva participated in the focus group discussion, Gloria’s prior experience had a positive impact on their persuasion and eventually played together. Initially, Peggie’s social activities are heavily influenced by Gloria. Gloria always takes care of Peggie, who is not interested, and tries to socialize together. Peggie had a very negative attitude towards the uDraw system at the beginning.

*“I am so tired, you know that this is my nap time. Can I go back and take a nap?”*

We were able to see her gradually growing through Gloria and gradually enjoying using the uDraw system. Older adults typically learn from their relationships about new products and activities, which may influence the perceived benefit of new technology. Playing games together is a meaningful social activity for them. Peggie frequently mentions how positively her best friend Gloria affects her life during an interview.

*“I like to spend time with my girl (Gloria), If I’m feeling down and out, I just come here and meet my best friend Gloria who’s going to make me smile, or laugh, or whatever. I bet we’d make each other happy. Yeah, we communicate really well.”*

Our study points out that social value is important in encouraging older people to use technology. The benefits of engaging in using technology do not only include the learning of technology but also the engagement into a small group class, which can become an integral part of the social contexts of older adults.

### 5.3. Familiarity 

#### Keeping a Familiar Lifestyle for Older Adults Instead of Forcing Older Adults to Use Technology for Convenience

Older adults are likely to adopt interactive technology to maintain their independence and convenience at the end of life. That is, they do not exclusively rely on caregivers to do all their activities of daily living (ADLs) [33,34] and live more conveniently at their homes [67]. In this study, we found that older adults are reluctant to change their daily routines or old habits to pursue convenience by using technology. Older people can be engaged more and can adopt technology into their lives when technology is integrated into their lives without interfering with older adults’ basic routines. For example, Eva, Peggie, and Gloria use flip phones. Gloria shares information with her friends in a face-to-face manner rather than using technology. When being reminded about the upcoming events at the community center, they prefer to use the old-fashioned way.

*“Ok, can you write the day and time on the paper for me? I will mark it on the calendar when I go home.”*

From Eva’s quotation, we can see that she does not use technology unconditionally because convenience is enhanced by using technology. Eva has mentioned once that she recognizes that using a calendar app can effectively manage schedules, but it is her old habit to write appointment dates on physical calendars. The participants did not feel the need to learn to use technology until they broke the way they’d been using it. Eva mentioned during the focus group discussion that she never forgets taking medication or an appointment at the hospital. In this case, Eva will not purchase a smartphone to use the reminder app. She will also not ask someone to download the app or spend time learning how to use it. Rather, it is more important for Eva to develop the ability to explore the technology without fear by familiarizing herself with the existing functions of her current mobile phone so that she can use them to her benefit. Therefore, we need to consider ways to help older adults become familiar with the technologies that are widely and easily found near their living environments. 

### 5.4. Cognitive Activity 

#### Providing the Opportunity for Cognitive Activities with Technology Rather Than Physical Activity 

Staying physically active is important for older adults to use technology [3]. Many research and innovation in technology development for older adults specifically focused on health-related technology [68,69]. There are many ways in which ubiquitous and mobile technology can motivate people of all ages to be more physically active [35,36]. We found that older adults who participated in our study are more proactive when faced with cognitive challenges. They lose interest when they realize that they will not be able to overcome their physical issues. 

We introduced two kinds of Wii games to our research participants. *Wii play* games require the use of physical ability, and *Wii brain* games require the use of the mental ability. In order to play *Wii Play*, older adults need to move fast. For example, while playing a table tennis game, Eva cannot keep up with the speed of the game because his body movement is slow. Additionally, although it is much more advantageous to stand and play the game than to sit on a chair, she feels a burden on standing for a long time. She has a very difficult time maintaining a normal rally in the table tennis game due to physical limitations. In this case, we can observe that she loses her confidence quickly and is reluctant to try other mini games. However, when playing the *Wii Brain* game, we can observe the changed attitude of Eva. If the time limit does not solve the assigned problem, she expresses the desire to challenge again. Besides, after the focus group was over and all discussion sessions were over, Eva remained for 30 min to continue playing. We found out participants are more engaged in playing cognitive games. The following is Eva’s reaction after playing *Wii Play*.

*“You have to practice like anything else. We can do it if we practice. it’s good, it’s interesting, but sorry baby, this game is not for me”*

However, Participants in the Focus group responded much more positively to the *Wii Brain* game. We learned that our participants are more likely to do cognitive activity than physical activity.

Michael: *“Mentally. Yeah. Give my brain a workout. We don’t mind being challenged because we are challenged to everybody. It’s (Wii Brain) a little more exciting than that other one (Wii Play). It’s a fun activity, more have fun”*

Through this case, we learned that we need to pay attention to how the attitudes of the participants change passively in physical activity and actively in cognitive activity. When performing cognitive activities, older adults show a willingness to overcome their limitations, and they become interested because of the sense of accomplishment they feel when they complete a task.

### 5.5. Peer Support 

#### Providing Peer Interaction and in-Person Training When Introducing New Technology to Older Adults Rather Than Organizing Professional Group Training

Generally, many researchers believe that some of the difficulties that older adults face while using technology can be resolved to some extent through early training or education [37,70,71]. However, older adults need constant help until the interaction is over. Based on training experience through this study, group training unified to older adults is not suitable. While each individual uses the same system, their desired functions are clearly different. Providing training that satisfies an individual’s expectations may increase the usability of technology and improve engagement.

*“Once you (facilitator) leave with that, I’m not going to think about that anymore. That’s it. I’m going to learn this time, and the next time you come back, I’m going to know how to do it then”*

Additionally, most older adults do not trust new technologies. Several studies have concluded that the mistrust is due to the lack of previous experience with technologies [28,72]. This is an additional burden on older adults. Our participants are likely to commit their effort in learning or using the technologies [29]. In this situation, we found that peer support is needed to deliver the right information and to improve trust about technology to older adults. Bob mentioned that he finds himself far more comfortable when asking for help from friends within the Pakistani immigrant group rather than seeking assistance from their children.

*“I have a friend who uses the phone more than I do, so he’s the one that actually taught me how to use the feature to do a video phone call. Yeah. So now, like I said, there are two groups that I’m connected with, actually there are three. So, we can video chat with each other. However, I don’t want to bother my son, he is working at the department store. He is really busy”*

A significant external factor that caused Bob to be positive about the use of technology was that his peer community was well developed. It also helped make his active attitudes more positive. 

### 5.6. Role of Grandkids 

#### Grandkids Are More Powerful to Encourage Older Adults to Use Technology Than Adult Kids

According to Courtney et al., [7] the family is the major determinant of whether an older adult would adopt a technology or not [39,40]. Many studies are conducted to reduce the burden on a family caregiver to take care of older adults [73,74]. Sometimes caregivers recommend the use of high-performance mobile technology for older adults for their convenience, but this can be a burden for them. Older adults feel sorry for the fact that children should pay a high price for devices, they have to learn new features they are not familiar with, and they have to ask for problem-solving whenever a problem arises. We found that the greatest stimulus for older adults emotionally is the participation of grandkids. In the case of the uDraw tablet, GrandKids are often referred to as the person they want to play with.

*“I’d actually like to bring my grandkids, one of them. To see what it would be like to have them and we all try to draw. You can leave some messages to your grandkids?”*

The commercial focus needs to change from caregiver to grandkids. It is important to consider how to increase the intergenerational connection and how to increase the involvement of grandkids.

### 5.7. Use of Existing Features

#### Think a Way to Utilize the Existing Features According to the Needs of Older Adults Instead of Delivering New Ideas or Innovative Features

Much effort has been made to develop various innovative technologies for older adults for maximizing their physical, psychological, and environmental wellness [75]. Technological innovations have been set up for older adults such as high-tech wearable technology [41,42,43], voice-activated assistance [76,77], smart appliances, and even home robots [21,78]. However, we found that instead of creating innovations, it is better to integrate new features into the technology that is already in existence. In the case of John, he uses the mobile phone almost every day for repairing his old car, using it as a magnifier for small-size texts supported by the zoom-in function. Instead of looking for an innovative feature, John explored a basic feature and identified a way to meet his needs.

*“Like right here you got electrical, my clubhouse, I can take a look and see how the wires go and what wires go where, so I can hook it up better. I used it for that type of thing. Yeah, so I can read it. And it helps me when I’m working.”*

Another example as shown in the case of Michael, it is more effective to specify one familiar function more than to provide various functions of different kinds to older adults.

*“See? That’s what I am talking about. I just need a simple, simple thing. I don’t need all of these brushes, I don’t need all of these options. Please get me a simple palette and canvas, that’s it”*

Even though there is a certain function of interactive technologies that improve the quality of life of older adults and support their daily activities more smoothly, we should be wary of putting older adults in the digitized context where older adults should learn something new continuously to adopt the technology. Rather than continuing to create new functions, it is necessary to consider in advance how to use the existing embedded functions of mobile phones or computers that are easily accessible to older adults.

### 5.8. Awareness 

#### Make Older Adults Aware of the Opportunity to Use Interactive Technologies That Can Be Enjoyed Free of Charge, Regardless of Cost

Another factor that influences technology adoption to older adults is affordability [44,45]. For those not connected to technology, the start-up costs (both the financial costs and the learning costs) are so unattractive as to not make the service interesting. Besides, older adults are less aware of the importance or significance of the technologies, and they are not willing to spend money on something they do not understand [79]. Before considering affordable prices for older adults to adopt technology, our study shows that it is more important to make older adults available more frequently to various technologies. In the case of Michael, he emphasized the importance of awareness in the in-depth interviews and focus group discussions. He said that he would like to use the system once he fully understood when and how uDraw was available, what features of uDraw he could use, and how he could use the system without difficulty with his abilities and experience. In other words, he was ready to engage in something new and was interested in trying it, but he did not know the options available to him. Older adults thus need to be fully informed in advance before they attempt to use the system.

*“I would love learning new things, but I have never had the opportunity to actually learn.”*

The aging population is generally not aware of the new technologies and their utility. Therefore, there is a critical need for older adults to be enlightened about the new technologies that are potentially useful for their ADLs, since lack of awareness is a barrier to adoption. Older people think technology is expensive, so they are reluctant to try technology because they are afraid to pay for what they use. The more they do, the older they lose the chance to experience technology. Government programs should increase the chances of older adults accessing new innovative technologies for free in a community or senior centers. This process raises older adults’ interest and pleasurable experience in technology and becomes an opportunity to reduce the initial barrier to technology.

### 5.9. One off Benefit

#### Focus on One Time for Fun Instead of Providing Continued Benefit to Use Interactive Technologies

Many studies focused on older adults have also identified usefulness as a key factor [80,81]. Studies have shown that older adults are attracted to technologies that provide utilities that are clear and are deemed to improve their current wellbeing [28,82]. Generally, researchers believe that regardless of the novelty of a technology or its popularity, older adults are more likely to adopt that which they perceive to have a potential benefit or might help them attain their desired convenience [28,75]. When designing technology for older adults, designers and developers consider sustaining the use of technology for older adults. However, our study shows that older adults have identified one-off usefulness or benefit as an important value. Participants mentioned that the uDraw system is not a system to be used continuously because our participants are not interested in drawing but are satisfied with using the uDraw system to enjoy time with friends. The following is a quotation showing their view of the uDraw system.

*“So it’s something not going to use for a long time, it is just going to be for one time for fun, It’s just something that relaxes us, that we just enjoy doing together. That’s all.”*

In the use of the uDraw system during the research period, our participants appreciate the opportunity to have time for fun, relieve stress, and connect to other residents. They do not want to use the system for a long period of time. Helping older people to have positive feelings about technology through one-time interests can also be effective in promoting the engagement of older adults in the use of interactive technologies. 

## 6. Discussion

We present five case studies and identify nine factors that influence older adults’ behaviors and engagement with two given systems. In this section, we present a new research direction on the topic of older adults: engagement. Then, we suggest a methodological direction for studying older adults to understand the context of aging in a digitized world. 

### 6.1. A New Research Direction on the Importance of Initial Engagement

We claim that initial engagement is more important than need and usability and has different challenges for older adults based on their behavior with interactive technology. When designing interactive technology for older adults that are passive towards technology, consideration of the factor of initial engagements should precede the design of interactions or evaluation of usability issues in the early phase of interaction. In this study, we see that studying older adults’ engagement in the use of technology is limited, and often used interchangeably with the notion of usability or user needs. Our understanding of the notion of initial engagement and need is that they are both cognitive processes of the user, but usability is a physical process that is more related to actual usage. Usability becomes critical and encompasses a lot of what the user feels after they decide to use the system. To distinguish between initial engagement and need, we need to understand what older adults want versus what older adults need. Older adults would be more engaged in using technology when they want rather than the technology that designers think they need. For example, wearable technology may be a “required” system for older adults, but it might not be the system “wanted” by older adults. Since the initial engagement is part of user experience and helps significantly the user has a more positive experience. We should understand that initial engagement is as important as usability and user needs. More research is needed to distinguish the difference between initial engagement, usability and needs. Initial engagement values that we present here are not completely new, but these values deserve more attention when considering the older adults’ user experience of technology. These findings serve as a focus to inspire new research directions for future Human Computer Interaction (HCI) research. 

### 6.2. A Methodological Direction for Studying Older Adults’s Use of Technology

In this study, we experienced difficulties in conducting research with older adults in the context of technology. The difficulties are centered around recruiting enough participants and gathering sufficient data to generalize the results. While laboratory based studies make it easier to collect large amounts of data, and allow generalizations and significance testing, we learned that gaining insights from older adults in sufficient numbers in ethnographic studies is not reasonable or possible. Qualitative data, collected using the ethnographic approaches we adopted in this study to understand individual behavior, produce deep and valuable insights to understand the participants’ engagement in the use of technology. Observing and evaluating the use of technology by older adults in an artificially created research setting can yield results that differ from the patterns used by older adults in real life situations. In our study we noticed that the older adults behaved differently when they knew they were being observed (for example in the focus group sessions) and when they were engaging with the technology on their own when the researcher was not present. By observing older adults’ use of technology in their own community over 3 months, we were able to better understand and critically consider their responses and engagement with the technology interventions. This does not mean that ethnographic qualitative approaches are the only way to collect data about engagement with interactive technology, but more ethnographic studies are needed to comprehensively and holistically engage with older adults. 

## 7. Future Work

There are two distinct avenues for future research. First, as a theoretical concept, the engagement factors can be used to measure older adults’ initial engagement and to design engaging user experiences for older adults. We could test which factors directly affect older adults and increase their level of engagement with technology in the behavioral patterns of older adults, the use of technology, and cognitive aspects. Initial engagement has a huge influence on the mindset of older adults when they encounter new technology. Initial engagement is a phase that can be measured by time or by cognitive changes. Another research direction is to study the transition from initial engagement to regular use as a cognitive marker that indicates older adults have demonstrated certain behavior and cognitive changes when initial engagement changes to long term engagement. 

Second, as a practical tool, the engagement factors can be used to evaluate engagement with specific applications. Due to the COVID-19 pandemic, older adults may be required to use technology to receive care that is related to health and survival or communicate with a loved one in a remote setting. One of the application areas of interest for future research is smart home devices and smart services for older adults. Therefore, we can study the effectiveness of initial engagement by older adults in different application domains. 

## 8. Conclusions

Although older adults who acquire digital literacy are increasing, a significant number of older adults are still unable to initiate technology on their own without help. Therefore, we should not overlook the initial barrier of older adults in the use of technology, and more research should be conducted on how to overcome the initial barrier. Through a synthesis of past research, and a mixed method study with two technological interventions, this study provides a focus on understanding older adults’ initial engagement that can change older adults’ behavior toward technology from passive to active. Then, we identify the key factors that influence the initial engagement of older adults: Desirability, Social, Familiarity, Cognitive activity, Peer support, Role of grandkids, Use of existing features, Awareness, and One-off benefit. In terms of the research methodology, we presented an approach for a more comprehensive understanding of older adults when they face a new technology in the real context rather than conducting experiments and analyzing results based on a limited exposure and assessment focused on physical and cognitive characteristics. The initial engagement factors contribute to both researchers and practitioners working on the topic of older adults’ engagement, adoption and use of technology, as well as those investigating ways to design and develop engaging technology for improving quality of life at old age.

## Figures and Tables

**Table 1:** Interview Questions used for qualitative study.

Interview Questions
Positive Affect: Can you explain how you feel while using feature X? Comfort: Are you comfortable when using feature X? Feel involved: How helpful do you think feature X is for your social interaction? Perceived benefits and usefulness: How much do you think using feature X improves the quality of your life? Control: How easily do you think you can control feature X? Help: How much do you think you need the support to be able to use feature X? Discoverability and Learnability: How easily do you think you understand feature X? Persistency: How frequently do you use feature X?

The first question asked in the interview: What do you like the best about your cellphone? X in follow up questions is based on individual’s responses.

## Data Availability

Data collected during this study is not available due to privacy issues. The detailed nature of the data collected during the ethnographic study means that the data cannot be edited to eliminate the possibility of re-identification.
